# Good practice for conducting and reporting MEG research

**DOI:** 10.1016/j.neuroimage.2012.10.001

**Published:** 2013-01-15

**Authors:** Joachim Gross, Sylvain Baillet, Gareth R. Barnes, Richard N. Henson, Arjan Hillebrand, Ole Jensen, Karim Jerbi, Vladimir Litvak, Burkhard Maess, Robert Oostenveld, Lauri Parkkonen, Jason R. Taylor, Virginie van Wassenhove, Michael Wibral, Jan-Mathijs Schoffelen

**Affiliations:** a Centre for Cognitive Neuroimaging, University of Glasgow, Glasgow, UK; b McConnell Brain Imaging Centre, Montreal Neurological Institute, McGill University, Montreal, Canada; c Wellcome Trust Centre for Neuroimaging, UCL Institute of Neurology, London, WC1N 3BG UK; d MRC Cognition and Brain Sciences Unit, Cambridge, UK; e Department of Clinical Neurophysiology and Magnetoencephalography Center, VU University Medical Center, Amsterdam, The Netherlands; f Radboud University Nijmegen, Donders Institute for Brain Cognition and Behaviour, Nijmegen, The Netherlands; g Lyon Neuroscience Research Center (CRNL), INSERM U1028, CNRS UMR5292, Lyon University, Lyon, France; h MPI for Human Cognitive and Brain Sciences, Leipzig, Germany; i Brain Research Unit, O.V. Lounasmaa laboratory, Aalto University School of Science, Espoo, Finland; j Dept. of Biomedical Engineering and Computational Science, Aalto University School of Science, Espoo, Finland; k INSERM, U992, Cognitive Neuroimaging Unit, F-91191 Gif/Yvette, France; l CEA, DSV/I2BM, NeuroSpin Center, F-91191 Gif/Yvette, France; m Univ Paris-Sud, Cognitive Neuroimaging Unit, F-91191 Gif/Yvette, France; n Brain Imaging Centre, Frankfurt, Germany; o MPI for Psycholinguistics, Nijmegen, The Netherlands

**Keywords:** Magnetoencephalography, MEG, Acquisition, Analysis, Connectivity, Source localization, Guidelines, Recommendations, Reproducible research, Spectral analysis

## Abstract

Magnetoencephalographic (MEG) recordings are a rich source of information about the neural dynamics underlying cognitive processes in the brain, with excellent temporal and good spatial resolution. In recent years there have been considerable advances in MEG hardware developments and methods. Sophisticated analysis techniques are now routinely applied and continuously improved, leading to fascinating insights into the intricate dynamics of neural processes. However, the rapidly increasing level of complexity of the different steps in a MEG study make it difficult for novices, and sometimes even for experts, to stay aware of possible limitations and caveats. Furthermore, the complexity of MEG data acquisition and data analysis requires special attention when describing MEG studies in publications, in order to facilitate interpretation and reproduction of the results. This manuscript aims at making recommendations for a number of important data acquisition and data analysis steps and suggests details that should be specified in manuscripts reporting MEG studies. These recommendations will hopefully serve as guidelines that help to strengthen the position of the MEG research community within the field of neuroscience, and may foster discussion in order to further enhance the quality and impact of MEG research.

## Introduction

Recent methodological and technological developments in the field of magnetoencephalography (MEG) have led to a new level of sophistication for performing state-of-the-art data acquisition and analysis. Thanks to the unique features of MEG data in combination with improved analysis techniques, a steadily increasing number of researchers have realized the potential of MEG to answer neuroscientific questions. These developments pose specific challenges, both to the methods developers and to the empirical scientists. Given the breadth of expertise required (time-resolved paradigm designs, multidimensional time series analysis, source reconstruction, statistical analysis, etc.), even experienced researchers in the field may find it difficult to keep up with new developments. New methods and technologies may have been introduced without rigorous testing, validation and comparison with existing techniques. In addition, the level of experience and the sophistication of data acquisition and analysis are highly variable, not only within but especially between groups, and there is often little room left for exchange of data, ideas and people across the MEG community. Similar procedures are often developed several times independently in different labs (or even in the same lab); sometimes with little awareness of existing procedures/toolboxes.

We realize a genuine need for the development of good-practice guidelines, the implementation of validated analysis pipelines and the sharing of practical knowledge across the MEG community. Such an endeavor could have several dimensions: documentation of best practice (recommended procedures for a certain type of analysis), quality control (how to ascertain that the analysis step was successful/meaningful), reproducible research (how to ascertain that the result of an analysis can be reproduced by the same or another researcher), report of results (recommendations for reporting MEG analysis and results suitable for peer-reviewed publications), and dissemination of source code implementing recommended analysis pipelines.

Achieving these goals requires substantial contributions from the MEG community, but we believe that the benefits are manifold: First, contributions to this concerted development, rather than isolated efforts, are more likely to bring long-term benefits. Second, contributions from multiple groups are likely to be synergistic, and avoid redundant efforts. Third, a concerted effort will foster development of the entire field by consistently raising the standards of MEG research. Fourth, the reproducibility of research will be facilitated. Fifth, a community consensus will enable a more unified presentation of the MEG community to other areas in neuroscience in general, and neuroimaging in particular. Sixth, it will help novices in the field to become productive in their MEG research more rapidly. Seventh, it will facilitate training and sharing of expertise and knowledge in the field.

Here, we take the first step^1^ in this direction and propose research guidelines for the acquisition and analysis of MEG data. These guidelines are intended to be taken as general recommendations and to provide a basis for further discussion and improvement by the entire MEG community. These recommendations may be useful as a guide for those new to the field of MEG by providing the novice with information and references to guide them through their initial MEG data recording and analysis (see, for example, recent guidelines for fMRI by Poldrack et al., 2008). In addition, they may serve as a basis for ongoing discussions and improvements of ‘best practice’ for MEG data acquisition and analysis. The paper also highlights common pitfalls, specific to MEG, which may confound the interpretation of results.

For the sake of clarity and conciseness, the methods are not described in detail and the interested reader is referred to more specific publications instead. Two publications are of more general relevance across the different sections of the paper. First, a recent textbook provides a comprehensive overview of the field (Hansen et al., 2010) and is recommended reading material. It differs in scope from this publication because it does not intend to provide concise guidelines or recommendations. Second, guidelines for using event-related potentials have been suggested (Picton et al., 2000). Several sections of this earlier paper are relevant to MEG researchers as well. Picton et al. cover topics related to experimental design, informed consent, etc. that are not repeated here. Similarly, sections related to filtering, artifact removal and averaging are partly relevant for MEG analysis as well. Overall, the present article is complementary to Picton et al.'s EEG guidelines, in that it focuses on MEG-specific aspects with several additional sections such as spectral analysis, source reconstruction, connectivity analysis and statistics.

This manuscript does not give recommendations related to hardware, software, data handling, participant questionnaires, or consent forms. It is also not meant as a recommendation to perform MEG analysis with all the stages described here or as an endorsement of a specific analysis (for example the analysis of event-related fields may not be meaningful for some data). The manuscript rather covers the most common analysis stages and the researcher must decide on the suitability of each stage for the respective project or may employ a different analysis not described here. In addition, these guidelines are intended for basic research, not clinical applications: timely with the present initiative, the American Clinical MEG Society has recently published the first set of guidelines for clinical MEG (Burgess et al., 2011).

The paper is divided into seven sections, each of which represents a separate stage in the data collection and analysis process. It begins with data acquisition and is followed by different analysis steps: data preprocessing, analysis of event-related fields, spectral analysis, source reconstruction, connectivity analysis and statistics. Each section provides a general background of the topic and concludes with two sub-sections: recommendations and caveats, and suggestions for reporting results in peer-reviewed publications.

## Data acquisition

### Background

Data acquisition is a critical component of a MEG study as it affects data quality directly. To ensure optimal data quality, the following elements need to be taken into account:

• Operating status of the system and set-up (sufficient helium level, optimum calibration/tuning of the sensors, proper functioning of the interference suppression system including magnetic shielding, sufficiently low ambient magnetic interference level).
• Preparation of the participant (removal of magnetic materials that may distort the measurements), explaining the task and giving instructions to avoid movements, eye-blinks and eye movements during the trials.
• Experimental design (e.g., allowing for sufficient number of data trials after rejection of artifacts).
• General acquisition setup (sufficiently high sampling rate and filter pass-band, recording of supplementary data such as electro-oculogram (EOG), electrocardiogram (ECG), electromyogram (EMG), eyetracking, digitized head shape).

The following section describes recommended procedures falling into these four categories, to optimize data quality directly or to improve performance of subsequent analysis steps such as source estimation.

### Recommendations and caveats

• Before the participant enters the MEG laboratory: We recommend that the condition of the MEG system is recorded by performing an “empty room” recording of about 2 minute duration before and after the experiment. These records are snapshots of the noise level (and their changes) at the time of measurement, allowing for the identification of problems with the system. We suggest simple spectral analysis and storage of original data and spectra for later comparison. These datasets allow monitoring the noise level and system status over longer periods of time. Tuning of individual sensors may be required for optimal performance. A log book is helpful to record and communicate the status of sensors and the system in general.
• Subject preparation: Simultaneous acquisition of EOG (horizontal and vertical) and ECG is recommended for several reasons:
  • Eye movements/blinks may be induced by experimental conditions, task and instructions in a non-trivial way (Picton et al., 2000). Recording the EOG allows for comparison of eye movements/blinks between conditions (see the section on Data preprocessing).
  • The magnetic field generated by the heart is several orders of magnitude stronger than the field generated by the brain and may contribute as a strong source of artifacts to the data recorded at the MEG sensors. ECG components in the data may have a detrimental effect on source reconstruction and confound the interpretation of connectivity estimates.
  • The complex spatio-temporal profile of the QRS-complex may lead to spurious (i.e. not brain-related) cross-frequency coupling in the data.
  • In general, both EOG and ECG components can have a detrimental effect on single trial analysis (e.g. by affecting single trial phase and amplitude estimates to a variable degree at different frequencies).
  • EOG/ECG signals facilitate artifact rejection and correction by acting as references.
  • In certain situations especially when investigating gamma-band activity, or during experiments involving limb/jaw movements it may be advisable to record muscle activity e.g. from neck or jaw muscles with additional surface electrodes since muscle activity shows as high-frequency signals in the MEG recording (Whitham et al., 2007). Similarly, such activity may lead to erroneous interpretations of source reconstructions.
• Identifying unsuitable participants: Suitability of the participant for MEG studies can be tested using the following procedure. The subject is placed under the MEG helmet and is asked to perform a few simple tasks while the operator observes the MEG real-time display: series of deep breaths for about 5 to 10 s, eye blinking when prompted by the MEG operator, opening and closing the mouth about once per second, also when prompted. Large signals associated with these tasks would indicate likely contamination of the subsequent MEG recording. The reader is referred to http://www.megcommunity.org for additional information such as instructions or questionnaires regarding subject preparation and instruction to minimize sources of artifacts. Degaussing techniques may be used, with reasonable chances of success, to reduce magnetization of dental work and other sources of magnetic interference.
• Placement of head localization coils and head digitization: It is recommended to attach coils on parts of the participant's head that are well covered by the sensors. Strictly symmetric coil positions should be avoided as they could create problems for automatic localization of the coils. Also, when a digitizer is available, the digitization of the head shape is recommended because this allows for an improved coregistration with an individual or standard anatomical MRI by means of surface based alignment (Whalen et al., 2008). Digitization should cover the whole scalp including points that mark the edges of the nose and/or chin, as this improves surface-based registration accuracy. Movements of the participant during digitization may compromise the quality of the head shape and the coregistration and hence should be avoided. If possible, the digitization procedure should be performed at a distance from large metal objects as these may affect the accuracy of the digitization.
• Positioning of the participant under the dewar: It is recommended to position participants as close as possible to the MEG sensors (e.g. by lowering the dewar or raising the chair until the head is in contact with the surface of the helmet). Moreover, making sure that the position of the subject's head is left–right symmetric and that it is not tilted around the anterior–posterior axis facilitates subsequent alignment of sensor-level data across subjects. It is advisable that the back of the subject's head rests either directly against the dewar or on a thin cushion on the helmet surface, depending on the desired head position for the study and the size of the subject's head. If possible, software which shows the head position with respect to the dewar immediately after activation of head localization coils should be used to ensure correct positioning. Additional mechanical head support can be provided to minimize head movements during the experiment; such movements are detrimental to data quality. If possible, head position should be recorded continuously to allow for offline control or correction of data but even then head movements should be minimized. Empirical evidence from online head position recordings suggests that on average the largest head movements occur downward within the first few minutes of the recording. To minimize this effect, it may be useful to provide arm support with a laptop table or armrests and to have the participant seated under the dewar for a few minutes before the experiment starts. This time can be used to attach peripheral electrodes, instruct the participant or have him/her complete training trials. Alternatively, recordings can be performed in supine position, which can be more comfortable for prolonged recording sessions, except when EEG is recorded simultaneously as occipital electrodes may cause discomfort.
• It is advisable to perform at least 2 minute ‘resting state’ recordings before and after the experiment with the subject fully prepared and positioned in the MEG system and all required equipment (microphone, camera, projector, etc.) switched on. This is valuable baseline data in the absence of stimulation and can also be useful to identify more subtle stimulation artifacts on the MEG signal.
• Data acquisition: The usual configuration is to have an anti-aliasing (low-pass) filter cut-off at 1/3 or 1/4 of the sampling frequency. Therefore, as a rule of thumb, the sampling frequency should be 3–4 times higher than the highest frequency of interest. When in doubt, always use a higher sampling rate. It is recommended to compute and display online averages and to monitor real-time display so that data quality can be assessed already during the recording; movement of the subject, declining vigilance, slipping attention, sudden environmental magnetic interference, for example, may significantly deteriorate the data.
• Anatomical MRIs: The acquisition of individual anatomical MRIs of participants in MEG studies is desirable if source localization is part of the planned analysis. Reasons for this include:
  • Individual surface-based volume conductor models can be constructed from the anatomical MRI, for example, by segmenting one or more surfaces associated with a marked change in conductivity (e.g., skin–skull or skull–CSF boundaries Wolters et al., 2006).
  • Individual anatomical MRIs can be used for (non-)linear transformation to a standardized MRI space (e.g., MNI, Talairach), to facilitate group statistics (see Statistics section).
  • Estimated source currents can be constrained to a cortical surface (see Source localization section), which can be approximated by a tessellated mesh of the gray-matter or white–gray matter interface obtained from a segmentation of the MRI (Fischl et al., 2001). A simpler alternative that may be sufficient in some circumstances is to warp a template cortical mesh to each participant's brain, based on the inverse of the normalization transformation (Mattout et al., 2007).
  • Individual anatomical MRIs can be used for more accurate coregistration of the functional information from MEG with anatomical information from MRI using surface-based alignment.
  • It can be helpful to place a small marker with high MRI contrast (e.g., Vitamin–E capsule) on the participants head before the MRI scan to unambiguously differentiate left from right later in the MRI slices. Use of a high-resolution (e.g. 1 × 1 × 1 mm) T1-weighted MRI volume (e.g. MPRAGE) is recommended, though for separation of skull–scalp boundaries (for more accurate forward modeling; see later), an additional PD-weighted image, derived, e.g., from a multi-echo FLASH sequence, can be helpful.
  • If an anatomical MRI cannot be obtained for each participant, it is recommended to digitize the individual head shape accurately. This shape can be used instead of the individual MRI to approximately align the subject's head to a template head (Holliday et al., 2003) to allow for averaging across subjects. The scalp points may also be used to warp a full, template MRI volume to the individual head shape (Darvas et al., 2006). Yet, MRI-based alignment to a standard brain is more accurate as it takes intracranial structures into account (e.g. brain, ventricles).

Whichever coregistration method is used, visual inspection of the alignment is highly recommended. Wrong or inaccurate definition of the landmarks (e.g. swapping left and right preauricular points) leads to incorrect results and affects subsequent source modeling. Similarly, automatic surface based alignment procedures may show small numerical errors despite obvious misalignments. Accuracy of MEG–MRI registration is important as head models are often based on the anatomical MRI and coregistration errors can therefore propagate into the head model and lead-field computations, which ultimately affects the accuracy of source reconstructions.

Finally, it is preferred that individual MRIs are acquired after the MEG session, as MRI scanning may often lead to a considerable increase of ‘magnetic noise’ from the participant and may negatively affect MEG data quality. The time constant for dissipation of this noise is unknown but empirical evidence suggests that negative effects on MEG data quality have typically disappeared within 3 days after a MRI scan.

### Reporting

The following is a checklist for the details that should be reported in a scientific publication. Again, we focus on MEG-specific aspects and assume that authors follow general scientific standards for reporting details about participants (recruitment, gender, age, etc.), informed consent, ethics, experimental procedures, etc. (see also Picton et al., 2000).

• Preparation of subjects (use of non-magnetic clothes, questionnaire to exclude metal artifacts).
• The essence of the instructions to and possible debriefing of subjects.
• MEG: system (vendor, model, version of the acquisition software) and sensor type(s), sampling frequency and pass-band (cut-off frequencies of the acquisition filters).
• MRI: system (vendor, model) and sequence, voxel size.
• EOG/ECG/EMG: type, number, position and impedance of electrodes, type of the amplifier.
• Head shape: system used for the digitization of the head shape.
• Head movements: Were head movements recorded? What was the maximum movement? If continuous recording is not available, the maximum difference between head positions before and after the run should be stated.
• Position of the participant: Was the participant in seated or supine position? How was the head of the participant positioned in the MEG helmet?
• For external stimulation and recording devices: State delays with respect to MEG triggers. For example a photodiode can be used to determine visual stimulus onset. Were the data corrected for the delay? Coregistration: Describe the coregistration procedure. Were individual anatomical MRIs used?

In case of surface-based matching, the typical number of digitized points should be stated, the optimization algorithm and its adjustable parameters, and a performance measure describing the goodness of fit.

## Data preprocessing

### Background

The main aim of this analysis step is the removal of unwanted data components that contaminate the signals. Although careful preparation and instruction of participants can greatly reduce artifacts, the data are typically contaminated with various interference components unrelated to brain activity. These components can degrade the accuracy of subsequent analyses and therefore the importance of appropriate preprocessing and artifact handling can hardly be overestimated. In general, artifacts can be classified into three categories:

• *System related artifacts*, due to SQUID jumps, noisy, broken or saturated sensors.
• *External artifacts*, arising from generators of magnetic fields outside the human body such as magnetic noise from power lines and other environmental noise sources (elevators, air conditioners, nearby traffic, mechanical vibrations transmitted to the shielded room).
• *Physiological artifacts*, caused by eye movements, eye blinks, cardiac and muscular activity, and head movements. The movement of magnetic objects or particles attached or implanted to the body may give rise to artifacts (e.g. eye make-up, hair spray, magnetized dental fillings).

Two general strategies are available for dealing with artifacts. First, one may simply identify data segments contaminated by artifacts through visual inspection, by means of an automatic detection procedure or a combination thereof. Subsequently, the contaminated data segments are excluded from further analysis. The second strategy uses signal-processing techniques to reduce artifact components that are not caused by brain activity while preserving signals originating from the brain. These artifact correction methods mostly make use of linear transformations or regression techniques applied to the sensor data (Ille et al., 2002, Parra et al., 2005, Schlögl et al., 2007, Wallstrom et al., 2004). Linear transformations (Gross and Ioannides, 1999) can be obtained from principal component analysis (PCA), independent component analysis (ICA) (Barbati et al., 2004, Ille et al., 2002, Jung et al., 2000, Makeig et al., 1996, Onton and Makeig, 2006, Parra et al., 2005, Rong and Contreras-Vidal, 2006, Vigario, 1997), through signal space projection (SSP) (Uusitalo and Ilmoniemi, 1997) or signal space separation (SSS) (Taulu and Simola, 2006, Taulu et al., 2004). The applicability of PCA, ICA and regression techniques relies on the assumption that the spatial topographies of the artifacts are stable across time, and that these spatial signatures can be described with a limited number of spatial components. SSS is implemented in Elekta MEG systems but could in principle be extended to all MEG instruments. Some MEG systems (CTF/VSM, 4D Neuroimaging and Yokogawa systems) record environmental magnetic noise from distant magnetic sources using an array of reference sensors. These additional signals can be used as regressors for cleaning the main MEG sensors using signal processing techniques similar to adaptive filtering. Some systems also provide the opportunity to use the signals from the reference sensors in order to create higher-order synthetic gradiometers with improved signal-to-noise ratio (SNR).

### Recommendations and caveats

To the best of our knowledge, the respective performances of all different artifact correction methods have not been compared comprehensively (but see Wallstrom et al., 2004, Parra et al., 2005, Ille et al., 2002). Consequently, our recommendations are based on the available literature and empirical evidence from the authors. Whenever possible, we recommend a procedure to validate the performance of an artifact correction step. In this section recommendations are sorted from strong to weak based on available evidence.

• Visual inspection of data is highly recommended for the identification of artifacts and for checking the accuracy of artifact correction methods. The identification of periodic artifacts (cardiac artifacts, line interference) can be improved by inspection of power spectra. However, note that the (often subjective) criteria used for visual inspection must be explained as formally as possible, so that the analysis can be reproduced.
• Dealing with *system*-*related artifacts*:
  • It is mandatory to reject flat and very noisy channels before proceeding to the next analysis step. Sensor-based analysis (especially if group analysis is intended) will likely benefit from interpolation of noisy/bad sensors to ensure the same number of sensors across participants; see e.g. (Knösche, 2002, Medvedovsky et al., 2007, Taulu et al., 2004). Validation: Topographies with bad channels excluded should be compared with interpolated topographies. Data epochs contaminated by SQUID jumps should either be removed, or the corresponding sensors interpolated if jumps occur frequently.
  • Line interference can be reduced by fitting and subtracting sine and cosine functions (Mitra and Pesaran, 1999) at the line frequency and its harmonics, or with ICA (Barbati et al., 2004). Consider using a comb filter or a series of notch filters when the amplitude of the noise is not sufficiently stable over the time window of interest (making the artifact slightly broader in frequency domain). Validation: Visual inspection or quantification of the attained attenuation at the line frequency and its harmonics; the required power spectra should be estimated from at least 30 s of data.
• Correcting for *physiological artifacts*:
  • The artifact correction techniques briefly described above can be used to suppress EOG/ECG artifacts. If external signals have been recorded (EOG, ECG, EMG), the identification of artifactual components may be facilitated. The extra signals (EOG, EMG, ECG) should also be subjected to the same analysis as the MEG signals (e.g. averaging or time-frequency estimation) to ensure that allegedly relevant cognitive components in the MEG signal are not also evident in the peripheral recordings (Picton et al., 2000). Validation: MEG signals before and after artifact correction should be compared. For example, the average of data segments time-locked to the external artifact before and after component removal can be compared to validate that the artifact was removed successfully. In addition, the average of data segments time-locked to the stimulus before and after component removal can be compared to ensure that artifact correction did not affect previously uncontaminated data.
  • Correcting for head movements: modern MEG systems can continuously monitor the head position (see Data acquisition section). Subsequent correction of MEG signals is possible using an inverse–forward projection (Knösche, 2002) or SSS (Medvedovsky et al., 2007, Taulu and Simola, 2006, Taulu et al., 2004). Validation: These methods may be unstable especially in the presence of large movements in the order of several centimeters and they may modify the MEG signals in a drastic way. Therefore signals before and after movement correction should be compared.
• Rejection of artifacts using automatic or semi-automatic methods, or based on visual inspection:
  • Semi-automatic methods are preferred, because it is often difficult to ensure robust performance for completely automatic artifact rejection methods.
  • Any artifact rejection method may lead to unequal number of trials across experimental conditions. This leads to bias in the estimation of some measures across trials, such as some connectivity measures (see Connectivity analysis section), in which case one could randomly select the same number of trials from each condition. Such equalization of the number of trials may not be necessary for other metrics such as the mean amplitude across trials in ERFs, for which parametric statistical methods exist to accommodate different numbers of trials and for which removing trials would only reduce statistical power.
  • Even if physiological artifact correction (as described above) is used, some trials may need to be rejected if, for example, a blink or eye movement occurs during presentation of a brief visual stimulus, precluding or adversely affecting its perception.
• The order of the steps is important. For example, SQUID jump artifacts will affect the performance of ICA or PCA methods due to the large variance of these jumps. In general, the following order is advisable:
• Removal of system-related artifacts. This step is independent of other steps and should be performed first. Noisy channels or squid jumps compromise the outcome of PCA, ICA and regression based methods, and also affects the application of digital filters (ringing).
• Removal of line noise.
• Removal of environmental noise. ICA, PCA, regression-based methods or SSS can be applied to remove environmental noise.
• Removal of physiological artifacts. In this step, EOG or ECG artifacts are removed.
• The removal of spatial components or subtraction of weighted reference signals from the data using subspace projection or regression techniques also needs to be taken into account in the forward model when subsequent source reconstruction is intended.
• Low-frequency modulations in the MEG signal across many channels can be caused by breathing-related artifacts (e.g. if participants do not wear MEG-compatible clothes). It can be attenuated using a high-pass filter applied to the raw, continuous recordings (about 1 Hz cut-off frequency).
• Stimulation devices, particularly electric nerve stimulators, can cause spike-like artifacts. Filtering these artifacts may yield signals that may be interpreted erroneously as ‘stimulus-induced oscillations’ or evoked components. Consider either applying a PCA event-related correction, or, if the artifact occurs before the expected brain response, cut out the contaminated segment and interpolate the omitted samples.
• Artifacts may have frequency components that fall within the range of physiologically relevant bands. Muscle artifacts (for example, from neck or jaw muscles) appear in the MEG signal as high-frequency oscillatory bursts.

### Reporting

• The method section describing the preprocessing of an MEG study should not only describe all parameters related to the respective preprocessing steps but also the order in which these steps were carried out.
• State the number of bad MEG sensors that were excluded during acquisition or analysis. Were the signals of bad sensors interpolated?
• For all filters, indicate the type of the filter, filter order, cut-off frequencies and direction of filter application (forward, backward, both).
• For ICA, describe the ICA algorithm, the input data to ICA, the number of components that were estimated, the number of components that were excluded and criteria for selecting these components.
• State the number of clean trials or data segments used for subsequent analysis.
• If trials or data segments were rejected, especially if based on visual inspection, describe fully the criteria for rejection (Picton et al., 2000).
• Indicate whether and which head movement compensation algorithm was applied.
• For adaptive interference suppression methods, such as the temporal extension of SSS (Taulu and Simola, 2006), indicate the relevant parameters. For tSSS, those parameters would be the duration of the correlation window and the correlation limit.

## Analysis of event-related fields

### Background

An enduring tradition of MEG signal analysis – inherited from decades of EEG research – is to analyze brain responses that are evoked by a stimulus or an action, by averaging the data about each event – defined as an epoch – across trials. The underlying assumption is that there are consistent brain responses time-locked and ‘phase-locked’ to a specific event (e.g., a motor act or a presentation of a stimulus). Hence, it is straightforward to increase the signal-to-noise ratio of these responses by averaging epochs across trials, under the assumption that the rest of the data is uncorrelated noise, i.e., inconsistent in time or phase with respect to the event of interest. This simple practice has permitted a vast amount of research using so-called event-related potentials (ERP) in EEG, or event-related fields (ERF) in MEG (Handy, 2005, Niedermeyer and Silva, 2004).

Once averaging has been completed, several measures can be taken on ERP/ERF deflections. Responses are defined as waveform elements that have a significant amplitude with respect to the baseline of the recordings, and they may be characterized in terms of e.g., relative latency, topography, amplitude and duration with respect to a baseline or a specific test condition. Once again, the ERF literature is immense and cannot be summarized here. Multiple reviews and textbooks are available and describe in great detail the specificity and sensitivity of event-related components (Hansen et al., 2010).

It should be noted that the traditional model underlying trial averaging that discards all ongoing background activity as noise is no longer tenable in the light of recent research. A large number of studies have demonstrated that the phase and amplitude of ongoing brain activity at the time of stimulus presentation significantly affects behavior (Laskaris et al., 2003, Mathewson et al., 2009, Ploner et al., 2006, Romei et al., 2012, van Dijk et al., 2008). Indeed, a recent TMS-EEG study provides evidence for a causal relation between ongoing brain oscillations and behavior (Thut et al., 2011). Our analysis and interpretation of MEG results should acknowledge the fact that brain responses are not simply driven by sensory input but rather that sensory input interacts with the dynamically changing state of the brain (partly reflected in ongoing activity) in a complex manner (Buzsaki, 2007). Some tools for this type of (single-trial) analysis exist already (Laskaris et al., 2004, Makeig et al., 2004, Pernet et al., 2011, Schyns et al., 2011). However, trial averaging is still more common than single-trial analysis; thus we discuss it in the following subsections but expect to see a stronger focus on single-trial analysis in future versions of this or similar documents. That said, averaging produces robust and repeatable signals of high SNR that have a proven clinical utility (for example the pre-surgical localization of sensory cortex).

### Recommendations and caveats

• Minimum number of trials: As with most experimental effects in neuroscience, there is inter-trial and inter-subject variability in the amplitude and latency of MEG responses. Even typical responses from the visual and auditory systems may be largely attenuated or even absent in some individuals. The early (< 20 ms) sensory responses are typically tightly phase-locked to the stimulus but require a larger number of trials than later event-related components due to their small amplitude. For instance, auditory brainstem responses can be detected with MEG however, as many as 16,000 trials were required in a study by Parkkonen et al. (2009). Mid-latency responses (20–200 ms) are usually considered to be phase-locked to stimulation and are efficiently revealed by epoch-averaging although even single-trial responses can be detectable. Long-latency responses (> 200 ms) are thought to reflect higher-order cognitive processes and these responses are more prone to inter-trial and inter-subject variability. The later responses are usually longer-lasting and averaging further emphasizes the long duration due to the variation of the latency of single responses. Time–frequency (TF) decomposition techniques help reveal induced modulations of oscillatory activity that may accompany evoked responses. Although computation of TF-decompositions requires considerably more effort, they provide valuable complementary information to the ERF approach (see next section). Theoretically, if brain responses were perfectly time- and phase-locked to the stimulus on top of additive Gaussian noise, signal averaging would enhance the SNR in proportion to the square root of the number of trials. We recommend investigating the stability of the obtained brain responses using randomization techniques such as jackknife and bootstrap methods (Darvas et al., 2005, Graimann et al., 2002, Pernet et al., 2011). Overall, the number of trials required to reveal effects of interest is a trade-off between a minimum that depends on the physiology of the brain regions involved (e.g., in terms of cell density, cell types and locations; see Attal et al. (2009) for a modeling perspective on this question) and a maximum that depends on how long participants can perform the task at the required performance level, maintaining a stable head position and avoiding eye blinks, etc.
• Inter-stimulus intervals (ISI): It is often advisable to introduce a random element into the ISI in event-related paradigms. One reason is to reduce effects of expectancy on evoked responses; anticipating the upcoming stimulus is known to modulate brain activity (Clementz et al., 2002, Mnatsakanian and Tarkka, 2002). However, in cases where predicting the stimulus type rather than its onset time matters, such temporal expectancy effects may not be important. A second reason for having random jitter in the ISI is to avoid aliasing or accumulation of periodic interference (such as line noise) in the average: by jittering the ISI over an interval of ~ 1/*f*, one can suppress non-phase-locked oscillations below frequency *f*. Note also that too short ISIs may lead to superposition of evoked responses from consecutive trials, which is desirable only when investigating steady-state responses (though again, random jitter can allow such overlap to be deconvolved); conversely, unnecessarily long ISIs reduce the total number of trials and hence the SNR for a given duration of the experiment.
• High-frequency oscillations and muscle artifact contamination: Particularly the gamma frequency range (> 40 Hz) is susceptible to contaminating muscular activity, such as phasic contractions or micro-saccades, which may also be task-related (Melloni et al., 2009, Yuval-Greenberg and Deouell, 2009). Therefore, these kinds of confounds must be ruled out by, e.g., EOG and EMG measurements.
• Application of filters: Data filtering is a conceptually simple, though powerful technique to extract signals within a predefined frequency band of interest. Applying a filter to the data presupposes that the signals of interest will be mostly preserved while other frequency components, supposedly of no interest, will be attenuated. However, not all digital filters are suitable for MEG/EEG analyses. Besides the frequency response, several other parameters are needed to characterize a filter; phase response, delay, stop-band attenuation, stability, and computational efficiency are few such parameters. The phase response of a filter is particularly important in MEG/EEG processing, and some filters may be inappropriate in that respect: generally, finite impulse response (FIR) and fast-Fourier-transform-based (FFT) filters can have a linear phase response (constant delay independent of frequency) whereas infinite impulse response (IIR) filters distort the phase in a frequency-dependent manner but they are computationally more efficient. The delay introduced by filters can be conveniently compensated for by applying the filter twice: once forward and once backward on the MEG/EEG time series. However, filters may exhibit “edge effects” at the beginning and end of the time series, and may require a large number of time samples when applying high-pass filters with low cut-off frequencies (as the length of the FIR filter increases). Hence it is generally advisable to apply digital filters on longer episodes of data, such as on the original ‘raw’ recordings instead of filtering the average. Note that filtering can lead to misleading results for causality analysis and should be applied only when the user is aware of the potential consequences (Florin et al., 2010, Barnett and Seth, 2011).
• Baseline correction: The raw MEG signals from the sensors typically have a large offset. This offset is there because SQUID behavior is periodic in applied flux and no zero level exists. In addition to this offset there will be some low-frequency noise from the SQUIDs and slow ambient field fluctuations. The correction for these low frequency effects is called baseline correction, which is very much akin to the typical EEG practice. The offset can be efficiently eliminated using a high-pass filter with a low cut-off frequency (< 0.3 Hz, with at least 100-dB DC attenuation) applied on the continuous raw MEG traces. Applying such filter on shorter epoched data would certainly cause edge effects and uncontrolled contamination of signals and is therefore not recommended. Another approach is to subtract the average value of the data across specific time intervals from the signal of each channel. The interval is defined as the baseline, and it should not contain event-related fields, while being close in time to the event occurrence. The baseline duration must be long enough for an accurate estimate of the DC value at each sensor. The required number of samples depends on the noise in the data so a range of values around baseline duration of about 100 samples may be explored.
• Group analyses of sensor-level data: Because the head is relatively free to move under the MEG array, grand (i.e., across subjects) or inter-run averaging is not encouraged in MEG at the sensor level without applying movement compensation techniques (Knösche, 2002, Medvedovsky et al., 2007), or without at least checking that only limited head displacements occurred between runs. However, trial averaging may be advantageously performed across runs on the source times series for each run, although the SNR of the un-averaged data that is used for the source estimation is lower in this case, which may be problematic in studies with a small number of trials per run. If inter-subject averaging is performed in the source space, typical geometrical normalization techniques such as those used in fMRI studies need to be applied across subjects, which are now a more consistent part of the MEG analysis pipeline and are featured in advanced software applications (Baillet et al., 2011). That said, raw sensor level analysis even without head position correction is an assumption-free analysis of the data and will generally err on the conservative (false negative) side (see the Statistics section).

### Reporting

• A clear chronology of stimulus presentation, featuring ISIs, stimulus types and durations.
• The number of epochs used in each experimental condition and for all subjects;
• Technique used for baseline correction (high-pass filtering; DC subtraction) and its parameters (filter type, baseline definition and window length, filter attenuation at DC in dB).
• Filtering: filter types, frequency cut-offs, filter order/length of filter kernel.
• Control of possible contamination of high-frequency (> 40 Hz) components from muscle artifacts.
• If applicable, mention the criteria used for the estimation of peak latencies, amplitudes and the time window of this analysis.
• If different sensor types were combined (such as magnetometers and gradiometers) specify the method used.
• If dimensionality reduction was performed (PCA, ICA, sensor selection) clearly state the procedure and the criteria for selecting components/sensors.

## Spectral analysis

### Background

The aim of spectral analysis is the transformation of signals into the (time-)frequency domain. This step is motivated by the fact that (transient) oscillatory signal components are more readily observable in the (time-)frequency domain. Also, oscillatory signal components have a more compact representation in the spectral domain. Researchers have a large variety of parametric and non-parametric spectral estimation procedures at their disposal. A comprehensive introduction and discussion is beyond the scope of this manuscript and we refer the reader to previous publications in the general field of spectral analysis (Brillinger, 2001, Flandrin, 1999, Percival and Walden, 1993, Stoica and Moses, 1997) and spectral analysis for electrophysiological data (Bruns, 2004, Mitra and Pesaran, 1999, Muthuswamy and Thakor, 1998). It seems that the choice of a specific spectral analysis method is less critical than its correct application. In fact, several of the commonly used methods (Fourier transform, wavelet transform, Hilbert transform) are very similar and can be described in a common framework (Bruns, 2004, Le Van Quyen et al., 2001). Consequently, we refrain from recommending a specific method but advise on some aspects of their use.

The researcher has to decide between parametric and non-parametric methods. Examples of non-parametric methods are the Fourier transform, wavelet transform or the Hilbert transform. The most commonly used parametric spectral estimation technique is based on autoregressive (AR) modeling (Astolfi et al., 2008, Schloegl et al., 2006, Seth et al., 2011). Although spectra computed from AR-models can potentially result in higher time and/or frequency resolution (Nalatore and Rangarajan, 2009) they are sensitive to user-defined parameters such as the model order.

A typical non-parametric time-frequency analysis involves the selection of a short segment of data with a sliding window approach, which is subjected to spectral analysis, for example using the Fast Fourier Transform (FFT) algorithm. Equivalently, this time–frequency transformation can be thought of as a convolution of the time domain signal with a set of windowed frequency-specific basis functions (wavelets; typically a sine and cosine wave of a specific frequency multiplied with a taper function, for example a Gaussian window). The different non-parametric methods differ mainly in their choice of tapers, i.e. the set of window functions with which the overlapping time-segments are multiplied to control for spectral leakage, and the use of fixed versus variable window length for different frequencies. Controlled frequency smoothing, independent of the spectral resolution determined by the choice of window length, can be obtained by combining spectral estimates corresponding to multiple tapers (Mitra and Pesaran, 1999, Percival and Walden, 1993).

### Recommendations and caveats

MEG researchers with little signal processing experience may find it easier to start spectral analysis with non-parametric methods. Therefore most recommendations in this section pertain to the non-parametric estimation methods.

• Zero-padding may be used to achieve spectral interpolation in order to be able to average the spectral representation of trials of variable length. Note that the spectral interpolation does not add information as such. It is recommended to perform a trial-based mean subtraction prior to the spectral analysis, in order to avoid potential spectral leakage effects introduced by zero-padding.
• In general one should beware of adapting the data window to the frequency under consideration (long window for low frequencies, short window for high frequencies) to optimize temporal resolution. Although this allows the number of cycles per window to be kept constant across frequencies it leads to unequal numbers of data points contributing to the time–frequency representation for different frequencies. This is not problematic as long as in any subsequent statistical test these changes in (spectrogram) image smoothness are properly accounted for using methods such as non-stationary random field theory (see later). To avoid this complication it is often advisable to use the same length of data segments for all frequencies, or to analyze ranges of frequency separately, e.g. use one window length for frequencies below 30 Hz and another (shorter) window for frequencies above 30 Hz.
• The bandwidth of the different frequency components in electrophysiological data increases with frequency. Also, across subjects, there is variability of the peak frequency (e.g. of the subject-specific gamma-band oscillatory activity) that is more pronounced for the higher frequency bands. For optimal sensitivity, it is therefore recommended to increase frequency smoothing at higher frequencies: for example, to apply little smoothing for frequencies below about 30 Hz (e.g., a few tapers implementing up to 2 Hz smoothing), but greater smoothing at higher frequencies, particularly above 50 Hz (e.g., multiple tapes implementing up to 10–15 Hz smoothing) (Hoogenboom et al., 2006, Schoffelen et al., 2011).
• The absolute power of MEG data is difficult to compare across sensors, sessions and participants. This can for example be due to inter-individual differences in neural activity and to differences in the position of the subject (e.g. differences in head size influencing the distance of the brain to the sensor array). Also, absolute power decreases with increasing frequency. For these reasons, to facilitate the interpretation of the spectral quantities, a frequency-specific correction is often applied using a baseline time-window or a baseline task. This correction is most conveniently expressed as a relative change. An alternative correction can be based on the difference in power levels, or the difference in the log of the power, which is equivalent to computing the relative change. Also, one could consider taking variance information in the baseline into account, and compute a z-score. Finally, one could compute the relative power, where the power in each frequency band is normalized by the total (broad-band) power.
• The selection of the time-window to be used for baseline normalization is a critical step. Generally, when choosing a baseline in the pre-stimulus period, one should avoid extending the baseline up to stimulus onset (t = 0) in order to avoid an overlap of baseline windows with post-stimulus data samples. Similarly, the baseline onset time should avoid including data segments affected by the end of the previous stimulus. Although − 50 ms or − 100 ms could be considered a sufficient upper bound for baseline offset and 300 to 500 ms a reasonable baseline duration, the optimal values depend on the paradigm and window length.
• A baseline shift close to stimulus onset may reduce the magnitude of low-frequency stimulus-related components in a time–frequency plot.
• The intrinsic frequency resolution of the non-parametric spectral estimate is limited by the length L of the moving window, and equals 1/L. The interpretation of the frequency of the peak of a band-limited effect needs to take the intrinsic frequency resolution into account.
• The frequency specificity of a wavelet may be compromised (i.e. leading to unwanted spectral leakage) by the exact implementation of the algorithm used. For example, when using zero-padding in combination with non-demeaned data, the artificially induced low frequency component may leak into the spectral estimates at higher frequencies. Therefore, examine the frequency domain representation of the wavelet to avoid erroneous interpretations, see Figure 1F in Box 2 in Tallon-Baudry and Bertrand (1999).
• The spectral estimate at a certain time–frequency point will be affected by data points prior to this time depending on the length of the wavelet or the data segment subjected to Fourier transform. Therefore, short transient artifacts in the time domain can affect spectral estimates at a later or earlier time.
• Inherent to the windowing technique is a loss of data at the edges of the trials. It is in general recommended to use a padding of the trials (with data) of at least half the window length of the lowest frequency wavelet.
• The MEG sensor type should be considered when interpreting results of spectral analysis. For example, a dipolar source is represented by the typical dipolar field pattern in MEG systems with magnetometer or axial gradiometer (a positive and a negative deflection on opposite sides of the source location). After spectral analysis this field pattern turns into two hot spots and may easily be misinterpreted as two active sources. Data recorded with (or transformed to) planar gradient sensors are easier to interpret because a dipole appears as a single local maximum in sensors just above its location.

### Reporting

#### General

• Length of time-window used.
• Amount of window overlap.
• Was normalization with a baseline estimate applied? If so, report which temporal interval was used, and how the normalization was done (e.g. absolute difference, relative change, z-score).
• Figures displaying the results should clearly describe any transformation applied to the data (e.g. log-transform). Each graph displaying values should provide a name for the values, a unit and a scale. In addition to images showing the effect size, consider displaying the results in a way that includes variance information (such as in a t-statistic).

#### Method-specific recommendations

##### Non-parametric methods

• Specify the amount of zero-padding applied.
• Specify the tapering strategy used to control for spectral leakage.

##### Parametric methods

• The algorithm used to estimate the AR-model.
• Order of the AR-model, and the method used for selecting the (optimal) model order.
• Clearly state all processing steps prior to estimating the AR-model (removal of ensemble mean, data normalization (e.g. by division with the ensemble standard deviation).

## Source reconstruction

### Background

The aim of source localization is to reconstruct the neural sources underlying the signals measured at the sensor level. The reconstruction can be broken down into a relatively uncontentious forward problem, which refers to the modeling of how magnetic fields propagate from the source space to the sensors; and the more contentious inverse problem that entails assumptions about how the brain works.

The M/EEG inverse problem is fundamentally ill-posed. In order to solve the inverse problem, it is necessary to employ prior knowledge (or constraints) in order to obtain a unique solution. We lack information for three fundamental reasons: First, we only have a limited number of measurements (sensors) compared to the potentially large numbers of sources. Second, certain current configurations will produce no measurable signals and we get no information on their presence (the so-called null space of the leadfields). Third, there are an infinite number of source current configurations in the space spanned by the leadfields that produce the exact same external magnetic field. These problems seem monumental but are analogous to the problems we encounter when interpreting a 3D scene from a 2D cinema film. Different source reconstruction techniques vary in the way in which they impose additional priors/constraints to arrive at a unique solution of the inverse problem by linear or non-linear computation. They vary with respect to the type and number of constraints used. Constraints might be that there are only a small number of sources active at a specific instant in time (multi-dipole models; Supek and Aine, 1993) or that the whole cortex is active to some degree but with the minimum energy necessary to describe the measured data (Hämäläinen and Ilmoniemi, 1994). It is beyond the scope of this paper to discuss source reconstruction algorithms in detail. For more information, we refer the reader to the relevant literature (Baillet et al., 2001, Hansen et al., 2010, Hillebrand et al., 2005, Ramirez, 2008, Wipf and Nagarajan, 2009).

All source reconstruction methods need a description of the forward model, i.e. a model that specifies the magnetic field distribution generated by a neural source with unitary strength at a known location and orientation. For the forward solution, a volume conductor or head model must first be defined. Typical volume conductor models are the spherical model with single or multiple spheres (Huang et al., 1999, Mosher et al., 1999) or spherical harmonics (Nolte, 2003); these can be elaborated on with boundary element models and finite element models (Dannhauer et al., 2011, Fuchs et al., 2007). The quality of the forward model and thus ultimately the quality of the source reconstruction will also greatly benefit from individual anatomical information, e.g. using the individual anatomical MRI to obtain a geometric description of the inner surface of the skull.

After defining the volume conductor model, a source model typically needs to be specified, which approximates the underlying continuous current density distribution with a finite number of parameters. Basic source models (such as dipole models or distributed source models) can be combined with orientation constraints (to exclude the radial orientation or restrict source orientation to be normal to the brain surface) and can be spatially restricted (e.g. to the cortex only rather than a grid covering the entire brain).

### Recommendations and caveats

• The quality of the source reconstruction will greatly benefit from an accurate coregistration and from the absence of subject head movement during the scan. Indeed, coregistration accuracy, which typically lies in the order of 5 mm (Whalen et al., 2008), and subject head movement may critically limit localization accuracy. Errors in coregistration will lead to errors in the reconstruction because the volume conductor (head) is shifted away from the correct position possibly causing a similar shift on the estimated source locations. When using sophisticated source models such as e.g. individual cortical meshes (López et al., 2012), or when using algorithms with high spatial resolution (Hillebrand and Barnes, 2003, Hillebrand and Barnes, 2011, Moradi et al., 2003) the influence of a poor coregistration may even be nonlinear.
• We recommend devoting extra attention to the placement of the head localization coils and to the specification of the fiducial locations during the preparation of the subject. Also, we recommend ensuring that the subject is comfortably positioned as this reduces movements during data acquisition. If high spatial accuracy is required it may be worth looking at improved head stabilization/coregistration procedures (Adjamian et al., 2004).
• In many cases, one is not interested in localization of sources per se, but in the extraction of source time series for further analysis (e.g. connectivity analysis). This can be done either as part of the source localization procedure or as a separate step. In the latter case it is also possible to specify source locations based on prior knowledge, i.e. using a region-of-interest (ROI) approach.
• In the process of reconstructing the time series at the source level, it is important to make sure that the linear weightings used to estimate a source time series not only pertain to activity at that location but also that they are unbiased. This means that, when comparing two conditions, both conditions should be included in the inversion process in order to get an unbiased linear mapping. By the same argument one should only compare estimates of neural activity within the same time–frequency window for which the inversion parameters were estimated. For example, in the case of a beamformer using a source covariance estimate based on the alpha frequency range will provide optimal suppression of other alpha generators, but these linear weights will not be useful to image effects in other frequency bands.
• When constraining the source reconstruction to a set of ROIs, or to an a- priori defined source model, brain activity that is not coming from the modeled areas may be erroneously projected onto these sources.
• Dimensionality reductions (e.g. PCA or ICA) need to be taken into consideration in source estimation. These steps effectively remove degrees of freedom from the data and reduce the rank of the sensor covariance matrix critical to the inversion algorithm.
• Estimates of spatial extent are difficult with MEG as the linear projections of the limited number of sensors are smooth and often data dependent. In such cases it can be useful to assess the point spread function (PSF) and/or cross-talk function (CTF), which are projections through the Resolution Matrix, which is the product of the forward matrix and the inverse operator matrix (Liu et al., 2002). The CTF for a given source, for example, is informative about the “leakage” of activity to a ROI from all other regions. Various summary measures of the PSF/CTF can also be helpful heuristics for evaluating the accuracy, smoothness and amplitude estimation in each ROI for a given inverse operator (Hauk et al., 2011). Spatial extent estimation generally requires specific algorithms using different source models (Cottereau et al., 2007, Hillebrand and Barnes, 2011, Jerbi et al., 2002). This is a critical issue that needs to be kept in mind and possibly acknowledged when interpreting the results based on a ROI approach.

### Reporting

#### General

• Details about the volume conductor model of the head (e.g. sphere, BEM, FEM) and algorithms used for the lead field computations.
• Rationale for choosing a particular source analysis method.
• Details about the source reconstruction algorithm and all its adjustable parameters, including the time intervals entered into the analysis and the choice of regularization and the rationale for this choice.
• Discussion of the particular shortcomings of the source localization technique used and how these may have affected results.
• Normalization procedures for spatial normalization after source localization.
• If coordinates are linked to brain structures: source of the lookup table (e.g. FSL atlas).

#### Method-specific recommendations

##### Beamforming

• Condition numbers of covariance matrices and metric used as beamformer output (e.g. noise-normalized power, pseudo-T, etc.; see Hillebrand and Barnes et al., 2005).
• If noise normalization is applied to the reconstructed source images, details about how the noise level was computed should be provided.
• Details with respect to the dipole grid that has been scanned: voxel size for a regular volumetric grid, or number of nodes for a cortical sheet.
• All details about the calculation of the covariance matrix should be specified (including the time window and filters or other preprocessing applied to the data).

##### Distributed source models

• Details with respect to the dipole grid that has been scanned: voxel size for a regular volumetric grid, or number of nodes for a cortical sheet, constraints to the dipole orientation.

##### Dipole fitting

• The solutions obtained with dipole fitting approaches depend heavily on choices that are made by the researcher (Miltner et al., 1994). Therefore, these choices should be described in much detail. In particular, alternative models should be fitted, and it should be explained why a reported solution was chosen over alternative models. For example, choices have to be made about the number of dipoles, the time windows that were used (single latency, multiple latencies), the exact dipole models that were used (moving, rotating, or fixed dipole), whether certain parameters were fixed while others were optimized. Principled and objective methods for this process exist (Kiebel et al., 2008, Supek and Aine, 1993). Also of importance are the cost-function and optimization algorithm that were used, e.g. cost-functions that include a cost for increased numbers of dipoles, as in the complexity term in various approximations to the Bayesian model-evidence (Kiebel et al., 2008). The stability of the solutions should be tested with respect to different initial guesses and with respect to noise, i.e. confidence intervals should be obtained, either through Monte Carlo simulations or through analytical/numerical approximations (Darvas et al., 2005, Fuchs et al., 2004).

## Connectivity analysis

### Background

The aim of MEG connectivity analysis is to investigate the information flow and interaction between brain areas (Ioannides, 2007, Palva and Palva, 2012, Schoffelen and Gross, 2009, Siegel et al., 2012, Stam and van Straaten, 2012, Jerbi et al., 2007). Modulations in connectivity through experimental manipulations will give insight into the dynamic routing of information in, and the reconfiguration of, brain networks. Here we will not focus on the large variety of possible connectivity metrics and their merits, but we will discuss some generic issues in MEG connectivity analysis. These issues require special attention with respect to data analysis, the interpretation of the results, statistical testing, and reporting.

As far as specific methods are concerned, the field of connectivity analysis in MEG seems far from reaching consensus on recommended methods. For each method, however, some degree of agreement for best practice seems to exist in the literature. As already mentioned, we will not discuss individual approaches extensively, but provide references to the relevant publications wherever appropriate. For a detailed overview, the reader is referred to Sakkalis (2011), Pereda et al. (2005) and Greenblatt et al. (2012).

The core issue that has to be considered when estimating connectivity in MEG-data is the issue of electromagnetic field spread. This term refers to the phenomenon that magnetic fields associated with electric currents extend to infinity. As a consequence, any neuronal current source generates a magnetic field that can in principle be seen by every MEG sensor, albeit with strongly differing intensity. This has a profound impact on the interpretation of the connectivity analysis results as most connectivity measures try to estimate whether two signals are dependent on each other. This dependency is then interpreted as a sign of a physiological interaction. With electromagnetic field spread, signals measured at the sensor level (and to a lesser extent also at the level of reconstructed source signals) are not independent. This leads to non-zero values for correlation (or most other functional connectivity metrics) between any two sensors, even in the absence of ‘connected’ underlying neuronal sources. As a consequence many connections may be spurious, and falsely be interpreted as being physiological (Schoffelen and Gross, 2009, Srinivasan et al., 2007). This even affects directed measures (Nolte et al., 2008), as well as nonlinear measures (Lindner et al., 2011).

One way to avoid incorrect interpretation of physiological interaction due to field spread is to quantify the connectivity that can be solely explained by non-instantaneous interactions. In measures based on phase differences, the instantaneous mixing due to field spread leads to a phase difference of either 0 or 180°. When the interaction is expressed as a complex-valued number effects of instantaneous mixing contribute only to the real part of the complex number. The imaginary part, e.g. the imaginary part of coherency, therefore quantifies interactions that cannot be explained by instantaneous mixing (Nolte et al., 2004). Another measure that explicitly quantifies non-instantaneous interactions is the phase lag index (Stam et al., 2007).

A recommended way to reduce the effects of field spread is to analyze the connectivity at the level of the underlying sources, rather than at the sensor level. Source-level connectivity analysis can be performed using either a one- or two-step approach.

Two-step approaches first perform an estimation of source time-courses. This can be done for example by computing a beamformer contrast to localize the regions of interest and then applying the beamformer filters for these locations to the sensor signals to obtain source time-courses. One-step approaches are based on a full model of MEG signal generation. Neuronal activity is generated using biophysical models of local cortical regions and their connectivity. Combining this with a physical model of MEG signal generation produces MEG signals that depend on parameters for the input to the brain (i.e. stimuli, experimental conditions) and the connectivity between brain areas. Similar to inversion schemes for source analysis this full model is then inverted, meaning that the model parameters are estimated along with a description of the model fit to the data. Implementations of this principle comprise DCM for EEG and MEG (Kiebel et al., 2009) as well as implementations that combine Granger causality analysis with state space models of brain activity into a one-step approach (Cheung et al., 2010).

Once connectivity patterns have been established, the functional networks can be characterized using graph theoretical tools (Bullmore and Bassett, 2011, Bullmore and Sporns, 2009, Ioannides, 2007, Stam and van Straaten, 2012).

### Recommendations and caveats

• Connectivity analysis should ideally be performed at the source level (Gross et al., 2001). One important reason is that the interpretation of the estimated connectivity between brain areas is typically much easier than at the sensor level and can be related to studies from other fields of neuroscience such as studies of anatomical connectivity, invasive electrophysiology or connectivity analysis using fMRI. Another important motivation for reconstructing source waveforms and analyzing connectivity at the source-level is that this approach alleviates the interpretational difficulties introduced by the mixing problem caused by electromagnetic field spread (Schoffelen and Gross, 2009). Source reconstruction admittedly adds another level of complexity to the analysis and may even cause problems if applied incorrectly. However, when sufficient unmixing can be achieved, source-level analysis of connectivity is preferable over sensor level analysis.
• If connectivity analysis is still performed at the sensor level it should be noted that the effect of field spread depends on the MEG sensor type. For example, magnetometers have a rather diffuse and spatially extended sensitivity profile with large overlaps between neighboring sensors in contrast to planar gradiometers with a more focal sensitivity profile. Consequently, connectivity results between magnetometer sensors are expected to show higher artificial values as compared with planar gradiometers.
• It is recommended to perform connectivity analysis in source space by contrasting two experimental conditions (or pre-stimulus and post-stimulus time windows or one condition and surrogate data). This further reduces the effect of field spread (Schoffelen and Gross, 2009). Note however, that power differences between conditions and general differences in signal-to-noise ratio will affect field spread and may lead to spurious connectivity differences.
• If two conditions are compared (or pre-stimulus and post-stimulus time windows or one condition and surrogate data) it may be important to match the total number of data samples in both conditions. Connectivity measures are often biased quantities, which makes them sensitive to the number of data samples (or number of trials).
• When using connectivity measures that use an estimate of the phase of a band-limited oscillation (e.g. phase–phase or phase–amplitude coupling analysis) we recommend a analyze careful selection of sufficiently narrow frequency bands. This is because the phase of a broadband signal does not have a straightforward interpretation.
• The quality of the estimate of the phase depends on the signal-to-noise ratio of the data, or in other words: the phase estimation from a noisy signal is less accurate. Consequently, connectivity metrics based on phase synchronization are affected by signal-to-noise, even though the metrics themselves are independent of signal amplitude. A reported difference in phase synchronization should therefore be accompanied by a report on the signal amplitudes (Muthukumaraswamy and Singh, 2011).
• As any other index of brain activity, connectivity measures need statistical testing. Tests can involve the comparison of two conditions, a task versus a baseline period or a comparison of two groups of subjects, similar to what can be done for more conventional measures of brain activity. Connectivity measures differ in three important aspects however. First, most connectivity measures are biased estimators, i.e. their absolute value systematically depends on the amount of data (Maris et al., 2007) and on the signal to noise ratio of the data (Haag and Borst, 1997). This necessitates that sources of bias are accounted for before proceeding with a statistical test, or the statistical test needs to be insensitive to the bias. Second, with connectivity measures it is sometimes not intended to compare across conditions or subjects. Rather, one would directly want to test the hypothesis whether a measured amount of connectivity is significant by comparing against artificially constructed (‘surrogate’) data, that share the same signal properties (spectra, etc.) but in which the connectivity has been destroyed in the construction of these surrogate data (Palus and Hoyer, 1998, Pereda et al., 2005). Last, multiple comparison problems are typically more severe for connectivity analysis, due to the large number of potential connections that is investigated, which is on the order of the number of signals squared. An advantage of regional source space analysis techniques over sensor level analysis here is that the number of source signals (of interest) is potentially much smaller than the number of available sensor signals.
• The creation of surrogate data to test the significance of connectivity in a single condition may not be straightforward. If inappropriate surrogate data is used, spurious results could be introduced (Stam, 2005).
• The removal of spatial components or subtraction of weighted reference signals (see Data preprocessing section) introduces correlations between the main MEG sensors, which may confound the interpretation of subsequent connectivity results.
• When the estimate of the connectivity involves an autoregressive model or a set of embedding parameters for state–space reconstruction (Granger causality and derived measures, transfer entropy), misspecification of the model or parameters may lead to false positive results and connectivity in the reverse direction. Principled approaches for choosing these parameters exist and should be used where possible (Ragwitz and Kantz, 2002).
• The assessment of coupling using the imaginary part of coherency has some limitations. First, it is by definition insensitive to true physiological zero-phase coupling. Second, problems may also arise when two conditions are compared, because it is not trivial to interpret a change in the imaginary part of coherency. An experimentally induced modulation may reflect a change in the preferred phase difference between two signals, a change in the magnitude of the coherency, or a change in both. Third, two or more independent sources with different orientation may be so close together that they cannot be distinguished from a single source that evolves over time with more than one orientation component, e.g. a rotating dipole. In this case non-zero values of the imaginary part of coherency can arise. Fourth, related to the previous point, sources of artifact such as the MCG typically need to be described as a complex rotating dipole and therefore contributions of the electrical signal from the heart lead to non-zero imaginary coherence.
• Unobserved sources may affect the interpretation of the observed connectivity pattern. Common input from a third source could explain an observed direct interaction pattern.
• When using a two-step approach to estimate connectivity at the source level it is important to only analyze features of the source time courses that are properly taken into account by the reconstruction method used. For example, when using a beamformer approach in a certain frequency range, source time courses should also be reconstructed and analyzed in this particular frequency range only; when using evoked activity to localize dipole sources then the source time courses of these dipoles should only be analyzed for their evoked activity content. This said, two-step approaches offer the possibility to inspect the reconstructed source time courses and to analyze them further outside the scope of connectivity analysis.
• In directed measures (Granger causality measures, transfer entropy) a test of the observed dependency against zero-lag dependencies can indicate the presence of field spread, e.g. shift test for transfer entropy (Lindner et al., 2011).

### Reporting

#### General

• Provide exact details of the preprocessing steps, e.g. filtering, as application of filters can alter the outcome of connectivity analysis (Florin et al., 2010).
• Describe procedures that were used to deal with artifacts such as the heartbeat signal or eyeblinks.
• Report on the amount of data used to estimate connectivity. Several connectivity measures such as coherence or phase-locking value are biased, where the amount of bias depends on the amount of data (e.g. trials) used to estimate connectivity. Address this potential bias problem if the amount of data in two experimental conditions is different (Maris et al., 2007).
• Discuss the interpretation of the results taking potential effects of electromagnetic field spread into account. This is particularly important for sensor-level analysis.
• Related to the previous point, discuss the interpretation of a conditional difference in estimated connectivity in the context of conditional differences in signal-to-noise ratio and differences in source power. Report power changes between conditions.

#### Method specific recommendations

##### Autoregressive model based connectivity measures (Granger causality and derived measures, and transfer entropy)

Report on the details of the autoregressive model fitting procedure, particularly with respect to the choice of the model order (also check recommendations referring to AR models under spectral analysis).

##### Dynamic causal modeling

Motivate the choice of particular models considered for evaluation. For example, why were particular source regions included in the model space and why were specific connection schemes considered. There is a considerable volume of pre-existing literature on this topic and we refer the reader to a reference where guidelines for model selection can be found (Stephan et al., 2010).

## Statistics

### Background

The aim of statistical analysis is to quantify the reliability of features that are estimated from the data, in the presence of noise (where noise could refer here to noise in the MEG recording (see Data preprocessing section), but also, for example, to inter-subject variability). This quantification typically takes the shape of a probability (p-value), a likelihood (e.g. when comparing models) or a confidence interval. Usually, the quantification is followed by an inferential step, e.g. a decision to reject or accept a particular experimental hypothesis, or the acceptance of a particular generative model of the data. Essentially, many steps in advanced MEG data analysis pipelines carry uncertainty that may warrant quantification, for example to assess the effect of particular parameter values on the quality of a source reconstruction. Here we will mainly consider statistical issues related to hypothesis testing that address the neuroscience question that has motivated data acquisition and the experimental design (Guilford and Fruchter, 1978).

The key questions can be posed using a variety of tests: parametric or non-parametric statistics, univariate or multivariate, and based on classical or Bayesian inference. These tests could be at the sensor or source level and have to take into account the control of error rate over multiple statistical tests.

A statistical approach is parametric when it relies on assumptions about the probability distribution of particular parameters of the data, for example the mean and variance of Gaussian distributions. Parametric statistics have been and still are extensively studied in the fMRI and PET communities – and recently adapted to EEG and MEG (Kiebel et al., 2005) – and popularized with software toolboxes such as SPM (Friston et al., 2007). Non-parametric approaches do not rely on such assumptions, but rather use resampling strategies to estimate probability distributions or confidence intervals from the data (Gross et al., 2003, Nichols and Holmes, 2002, Pantazis et al., 2005, Pernet et al., 2011, Singh et al., 2003).

Classical statistical inference relates to testing a formal hypothesis about the data, typically a null-hypothesis stating that an experimental manipulation has no effect on a particular feature in the data. Bayesian statistics can provide a probability of an effect being of a certain size, but require a prior probability distribution.

Multivariate versus mass univariate tests refer to whether multiple features (variables) are tested at once in a single test, or whether each variable is tested with a separate single test. When variables represent continuous values along one or more dimensions (e.g., two dimensions of a sensor topography, or of a time-frequency decomposition), multivariate tests can be used to identify significant patterns (modes) across dimensions (Carbonell et al., 2004, Friston et al., 1996, McIntosh and Lobaugh, 2004), while univariate tests can be used to localize significant effects within that space. In general terms, multivariate versus mass univariate approaches trade statistical sensitivity (power) against localization accuracy (in time or space), with multivariate tests usually having greater power but less localizing ability (assuming that there are sufficient replications relative to the number of variables). Multivariate tests can suggest networks of active regions within source space, or of cross-frequency dependencies, whereas mass univariate tests are necessary to identify whether an effect occurs in a specific region or specific frequency.

An important issue when doing statistical inference with a mass univariate (or multivariate) approach is the multiple comparison problem (MCP); the fact that the probability of incorrectly rejecting the null hypothesis increases with the number of tests. One approach is to control the Family-Wise Error (FWE), another is to control the False Discovery Rate (FDR). These approaches entail qualitatively different types of inference: FWE controls the false positive rate across all tests, FDR controls the false positive rate within the subset of tests declared significant (Genovese et al., 2002). There are both parametric and non-parametric methods to control the MCP. Non-parametric methods can estimate distributions of maximal statistics across tests by permutation or randomization (Maris and Oostenveld, 2007, Nichols and Holmes, 2002, Pantazis et al., 2005, Singh et al., 2003). Parametric approaches to address the MCP have been elaborated using the random fields theory (RFT) and have gained popularity through the SPM software (Friston et al., 2007). RFT takes into account the spatial/temporal correlations in the data resulting in a “corrected” p-value that is less conservative than a Bonferroni correction that simply divides the acceptable false positive rate by the number of tests. Furthermore, it provides a general framework that can be applied to any number of dimensions. The main advantage of the parametric approach is that it can easily accommodate complex statistical designs such as multiple regression or full factorial design. The main disadvantage is that there are distributional assumptions. Non-parametric methods for MCP have similar application to multiple dimensions, and offer a large range of test statistics, but they are limited to simpler statistical designs, primarily *t*-test and simple regression (Maris and Oostenveld, 2007).

In the context of statistics, it is a valid question to ask whether the statistical inference needs to be done in sensor or in source space. Indeed source estimates are often a linear combination of the sensor data, so any multivariate test that tests for linear combinations of channel data (e.g., Wilk's lambda) automatically provides a test of source tomographies (Carbonell et al., 2009). The distinction between sensor and source level analysis is analogous to that between non-parametric and parametric statistics. Sensor level analysis is relatively assumption free and robust, but given the assumptions (of the inversion) are correct, source level analysis will be more sensitive. Some specific situations where statistics might be better performed directly in source space are:

• When the sensor data are not aligned across replications (e.g. when the location of the head relative to MEG sensors differ across participants). Projection onto a common source space would address this issue through well-established techniques for spatial normalization (Ashburner and Friston, 1997, Fischl et al., 1999) although realignment of the data could also be done in sensor space (Knösche, 2002, Taulu and Simola, 2006, Taulu et al., 2004).
• When the source orientations vary substantially over subjects, for example in cortical regions like V1 it is likely that the same sensors even see polarity reversals over subjects.
• For multimodal datasets – for example, data from magnetometers, gradiometers and EEG – for which source space statistics after their combined localization (Molins et al., 2008; Henson et al., 2009) are likely to be more sensitive than statistics on each sensor-type alone.
• When there are priors that live in source space (e.g., from fMRI (Liu et al., 1998, Henson et al., 2010)), or via pooling of sparse priors across participants (Litvak and Friston, 2008), statistics may be more easily performed in source space.
• When inferences about specific brain regions are to be made.

A final issue concerns the level of generalization of inferences. To justify that a neuroscientific claim is true in a “typical” person, it can be sufficient to demonstrate that an effect is significant within each of a (small) number of participants (e.g., using an estimate of variance across trials for each participant). This “case series” approach is common in psychophysics and animal experiments. To justify a neuroscientific claim that an effect exists on average in the population as a whole and to quantify the variability of that effect across people, it is necessary to use sample-based (group-based) statistics. To extend inferences from a random sample to the population from which it was drawn, it is necessary to treat those observations (participants) as a random variable (a random variable is one that can have different values each time an experiment is run, as distinct from a fixed variable that has the same value for all instantiations of an experiment). For situations with multiple replications both across- and within-participants (e.g. across trials or sessions), this can be achieved by “mixed effect” (hierarchical linear) models, though in balanced designs (e.g. with equal numbers of trials for all participants), a simple summary measure (e.g. the mean) across trials/sessions is sufficient. Parametric statistics accommodate such sample-based inference easily, by virtue of their distributional assumptions; it is debatable whether permutation-based inference allows such generalization.

### Recommendations and caveats

• Decide in advance the key experimental comparisons of interest: e.g., across experimental conditions, and possibly within specific sensors/time windows/frequency windows. Avoid the issue of looking at the data before deciding what comparisons to test (a classic error being to inspect an evoked waveform to see when differences between conditions are maximal, and then choosing a window around those time points to perform a statistical test: in this case, multiple comparisons across time have implicitly been made, for which a correction is necessary).
• The decision to use parametric or non-parametric statistics should be based on examination or knowledge of the data distribution. For example, without transformation, distributions of power measures or phase-locking values are a-priori unlikely to be Gaussian, whereas the distribution of the mean field amplitude across trials is likely to be Gaussian across sufficient numbers of participants. While data examination is always desirable, it should be noted that formal tests of distributional assumptions are rarely sensitive unless there are sufficient replications, which may be a reason to prefer non-parametric methods when there are few replications.
• When the underlying assumptions are met (or the data are transformed in order to meet the assumptions), parametric methods will always be as or more sensitive than non-parametric methods. Conversely, non-parametric methods will be more robust when there are deviations from those assumptions. Non-parametric methods often estimate distributions from the data by permuting or randomizing data labels, though consideration must be given to the exchangeability of such labels (e.g., in the case of correlated observations, such as from successive timepoints). Non-parametric methods can be computationally expensive, though this is becoming less of an issue these days.
• Both RFT and non-parametric approaches to the MCP offer the ability to make inferences about specific features of test statistics: for example, not just the “size” of a statistic in each point in a space (or “peak” of a statistical field), but also its “extent”. Extent-level correction can be more sensitive than a height level correction at the cost of localizing power (e.g. Singh et al., 2003). This is why it is often used in fMRI, but its relevance to some MEG applications, such as sensor-time volumes, is questionable in cases like magnetometer data with their bipolar (non-contiguous) fields.
• Decide in advance whether the scientific question concerns the identification of spatiotemporal patterns or the localization and mapping of effects in space/time. This constrains the type of statistical model/tests that need to be applied. The former likely requires multivariate statistics, whereas the latter most likely requires a mass univariate approach.
• Multiple variables may still be modeled as factors within a univariate framework, for example in traditional analysis of variance (ANOVA) approaches for analyzing the mean amplitude of an evoked response across a specified peristimulus time window, in which each channel is a level of one or more factors (Picton et al., 2000). For a single channel factor, such an approach is actually equivalent to a multivariate test (Kiebel and Friston, 2004); though a potentially useful variant is to factorize channels according to a spatial dimension (e.g., left/right, anterior/posterior), providing some localizing ability (at least for variables like planar gradiometer magnitudes; the advantage of such an approach is less clear for magnetometer or axial gradiometer data).
• Justify assumptions about data distributions, theoretically and/or empirically. Check the distribution of data values (observations) before using any statistical test, e.g., for outliers, or to confirm distributional assumptions when performing a parametric test, or to check for correlations between observations when performing a non-parametric randomization test.
• Account for multiple comparisons — both across space/time/frequency, and across multiple contrasts within an experimental design. Make sure that the assumptions of any method for correcting for multiple comparisons are met, for example the smoothness of data for random-field models.
• Due to differential sensitivities of sensor and source space analyses it is sometimes the case that a particular effect is significant in one but not the other. When an effect is significant at the sensor level with all the proper corrections for multiple comparisons and the hypothesis is about the existence of an effect rather than about a specific area being involved, it could be acceptable to only report the peaks of a statistical map at the source level without requiring correction for multiple comparisons over the whole brain. When an effect is significant at the source level corrected for multiple comparisons and the choice of time and frequency windows for the source analysis can be motivated a-priori, a sensor-level test is not necessary. What should be avoided is doing a sensor-level test without proper MCP correction and using it to motivate a source-level test that achieves significance. This would constitute double-dipping (Kriegeskorte et al., 2009) similar to using peaks in the data to constrain a sensor-level test.
• Limiting the scope of statistical tests a-priori is a perfectly valid and powerful way to increase their sensitivity. Only resort to multiple tests for data dimensions about which you have no prior hypothesis and limit the number of these tests to the essential minimum. For example if your hypothesis is about an effect in the alpha band between 100 and 200 ms and it is likely that most of this window is affected, it would be better to average over the window, get a single number and avoid multiple comparisons altogether. If your hypothesis is about an increase in alpha power between 200 and 800 ms which is relatively brief but spans most of the alpha band it would be better to average over frequency and test one-dimensional power time courses only in the window of interest. Similarly, if your hypothesis is about, for example, the visual system, you can use a mask to limit your source level test to the visual areas.
• Clarify the type of inference you are making, whether it is based on FWE or FDR, or a random or fixed effects analysis of participants.
• Mass univariate methods in source space raise issues related to the appropriate way to deal with the MCP. Firstly, assumptions in methods like RFT may be violated. For example, RFT assumes a monotonic-decreasing spatial correlation in the error, but this is unlikely to be true for source-localized data because long-range correlations can be induced by the inverse operator (e.g. when sources are constrained to be normal to a gray-matter surface, in which case two dipoles that are far apart but with similar orientation can have more similar gain vectors than two dipoles close together but with different orientations). As a result, corrections to classical p-values using RFT will be too conservative. In this case, non-parametric MCP approaches may be preferable. An alternative is to use Bayesian inference, calculating a posterior probability of source strengths above a certain threshold for each point in source space. For example, using an empirical Bayesian approach with an implicit hierarchy over source voxels, “posterior probability maps” (PPMs) can be constructed (Friston and Penny, 2003). Provided posterior probabilities are interpreted as just that, rather than categorical statements that an effect is present or absent, then it can be argued that there is no MCP (Friston and Penny, 2003).

### Reporting

• Describe the full statistical model used: For example, describe the design matrix used in a (parametric) General Linear Model (GLM), report the degrees of freedom, and corrections for any correlation in the errors. For non-parametric tests, report the type of technique used (randomization, permutation, bootstrapping) and the number of resamples.
• Clarify what were a-priori and post-hoc comparisons, and the method for correcting for multiple comparisons.
• Report effect sizes (e.g. parameter estimates, such as means) as well as their statistics.

## Conclusion

In this manuscript we have attempted to lay down some basic guidelines for MEG analysis. Our motivation is to improve standards for MEG reporting and analysis, rather than to advocate a single analysis approach, and to unify and focus the MEG community. This is a difficult task, principally because after forty years of MEG development, it is one of the first papers of its kind. We realize that this can only be a first step that will hopefully lead to a lively discussion in the community and result in future consensus papers.

There are a number of exciting new areas of analysis, which have not been considered in detail here. These include methods such as dynamic causal modeling, single-trial analysis, multivariate classification and decoding techniques, as well as multimodal imaging.

What is clear is that the MEG data record has a rich information content. This is what makes MEG analysis more complicated than that of other techniques, but also means that the rewards are potentially much greater. VL and GRB are supported by core funding from the Wellcome Trust (091593/Z/10/Z).

## Footnotes

1 We envisage updates of this document based on comments from the community. Further information will be provided on http://www.megcommunity.org.
